# Advances in balance training to prevent falls in stroke patients: a scoping review

**DOI:** 10.3389/fneur.2024.1167954

**Published:** 2024-02-05

**Authors:** Kehan Chen, Siyi Zhu, Yidan Tang, Fuxia Lan, Zuoyan Liu

**Affiliations:** ^1^Department of Rehabilitation Medicine, Rehabilitation Medicine Key Laboratory of Sichuan Province, West China Hospital, Sichuan University, Chengdu, Sichuan, China; ^2^Department of Cardiology, West China Hospital, Sichuan University, Chengdu, Sichuan, China; ^3^West China School of Nursing, West China Hospital, Sichuan University, Chengdu, Sichuan, China

**Keywords:** stroke, rehabilitation, postural balance, exercise, accidental falls

## Abstract

**Objective:**

To summarize the status and characteristics of the available evidence, research gaps, and future research priorities for preventing falls in stroke patients through balance training.

**Methods:**

We used a scoping review framework. A systematic search of PUBMED, Embase, and Cochrane databases for main articles was conducted. Our study only included articles that on balance training and fall-related indicators in stroke patients. Two researchers independently screened the literature according to the inclusion and exclusion criteria. The data of demographic, clinical characteristics, intervention, sample, and outcome indicators were extracted. The characteristics and limitations of the included literature were comprehensively analyzed.

**Results:**

Of the 1,058 studies, 31 were included. The methods of balance training include regular balance training, Tai Chi, Yoga, task balance training, visual balance training, multisensory training, aquatic balance training, perturbation-based balance training, cognitive balance training, system-based balance training, and robot-assisted balance training. The commonly used outcome measures include clinical balance test, such as Berg balance scale (BBS), Timed Up-and-Go Test (TUG), Fall Risk Index assessment (FRI), Fall Efficacy Scale score (FES), and instrumented balance tests.

**Conclusion:**

This scoping review summarizes the existing primary research on preventing falls in stroke patients by balance training. Based on the summary of the existing evidence, the characteristics of balance training and their relation to falls in stroke patients were found. The future researches should explore how to develop personalized training program, the sound combination of various balance training, to more effectively prevent falls.

## 1 Introduction

Stroke is the second leading cause of death and the third leading cause of disability in the world (1). According to statistics, the global cost of stroke is more than US $721 billion (0.66% of global GDP), and the incidence of stroke (70%), mortality (43%), morbidity (102%) and disability (143%) are also on the rise (1990–2019) (1). The most common physical dysfunction in stroke patients is impaired balance, which has been shown in studies to have an incidence as high as 61–83%, and even in the chronic stage, the incidence is as high as 22–43% (2, 3).

Balance refers to the ability to keep the body in a state of balance, which can be divided into static and dynamic. Static balance is defined as the ability to keep balance in a position without moving, while dynamic balance is defined as the ability to keep certain positions during movement (4). The increased risks of falls, social isolation, and reduced physical activity were common in stroke patients with balance dysfunction (5, 6). Early identification and appropriate intervention can prevent balance dysfunction from becoming worse (5, 7, 8). A good balance is likely to be a rapid synergy between various physiological and cognitive factors to respond quickly and accurately to disturbances. This very complex system that can respond rapidly and accurately to prevent falls.

According to the World Health Organization, fall is sudden, involuntary and unintentional change of position, falling to the ground or a lower plane. Falling is a common complication after a stroke. Studies have shown that the incidence of falls in stroke patients is as high as 25–40%, and the injury rate is as high as 90, 32–83% of stroke patients are afraid of falling, and the risk of falling increased with the severity of stroke (9, 10). Falls lead to injuries, fractures, reduced quality of life, prolonged length of hospital stays (LOS) for stroke patients, and a heavy financial burden.

There have been many studies on balance training in stroke patients. Conventional balance training including sitting to stand, standing on one leg, using paralyzed and nonparalyzed limbs across stools of varying heights, standing on the bottom of foam or rocker, walking sideways, posture training on a therapy ball, reaching forward and side, standing with eyes closed, tandem standing, progression to tandem walking, lateral stepping, step forward and backward, walk forward, stomp up and down, throw and catch plastic balls (using soft volleyball) or small beanbags (11). Tai Chi and Yoga were ancient exercise, Tai Chi is effective in improving the balance function of stroke patients (12). In addition, water-based balance training, which is similar to land, is more efficacious (13). Reactive balance training (RBT) is a novel exercise designed to improve reactive balance control, its effect in reducing falls has been demonstrated in multiple studies. Perturbation-based balance training (PBT/PBBT) focuses on practicing responses to instability and aims to improve reactive balance control, reduce the risk of falls (14). PBT includes tasks that induce external perturbations, which are applied by external forces (e.g., pushing or pulling by a physical therapist), and internal perturbations include rapid movements that may cause loss of balance (e.g., balance disturbances during football playing, standing, and treadmill walking) (14). In recent years, balance training is also carried out through Wii Fit games, virtual reality (VR) (15), etc.

However, only a few studies included fall-related indicators in the outcome measures. To outline the scope and characteristics of any existing evidence on balance training for fall prevention, research gaps, and future research priorities, we conducted a scoping review to summarize and critically analyze the findings of all published articles.

## 2 Materials and methods

The scoping review was guided by the work of Arksey and O’Malley (16) and further refined by Peters Micah, initiated by the Joanna Briggs Institute (17). The Preferred Reporting Items for Systematic Reviews and Meta-Analyses extension for Scoping Reviews (PRISMA-ScR) guidelines were followed (18).

### 2.1 Search strategy

The search strategy was developed by two researchers and a science librarian in September 2022; Supplementary material 1 for details of the search strategy. Electronic databases including PUBMED, EMBASE, and Cochrane Library were systematically searched. The search strategy included a combination of MeSH Terms and other keywords. We combined the Boolean terms “or” (within columns) and “and” (between columns) to include all articles published from the beginning of each database’s creation to the search date (January 2023). Only articles written in English were included in this study.

### 2.2 Study selection

ENDNOTE X9 was used to manage the literature retrieved from the data and remove duplicate literature. The study had to be an intervention study based on balance training for preventing falls in stroke patients, and at least one fall related outcome index. Patients were excluded if they had balance training only but no fall prevention, if there were other types of training in the intervention besides balance training, or if they had fall prevention but not balance training. Scoping reviews have flexible study designs that allow authors to include any type of study that may be appropriate to answer their research question. We included any paper with a primary study design, such as RCTS and non-randomized controlled trials, case studies.

### 2.3 Data collection and synthesis

The characteristics of the included studies were extracted, including authors, publication date, study design, country/region, stroke type, gender, intervention measures, outcome indicators, etc. Before extraction, a data extraction table was developed, and sample articles were used for trial. Two researchers independently evaluated all relevant titles and abstracts according to the inclusion and exclusion criteria. In case of disagreement, the third researcher judged and revised and improved the table as needed. In the process, we also contacted experts in neurological rehabilitation for advice on developing eligibility criteria and to help us retrieve any relevant studies that may have been missed.

## 3 Results

**Figure 1:**
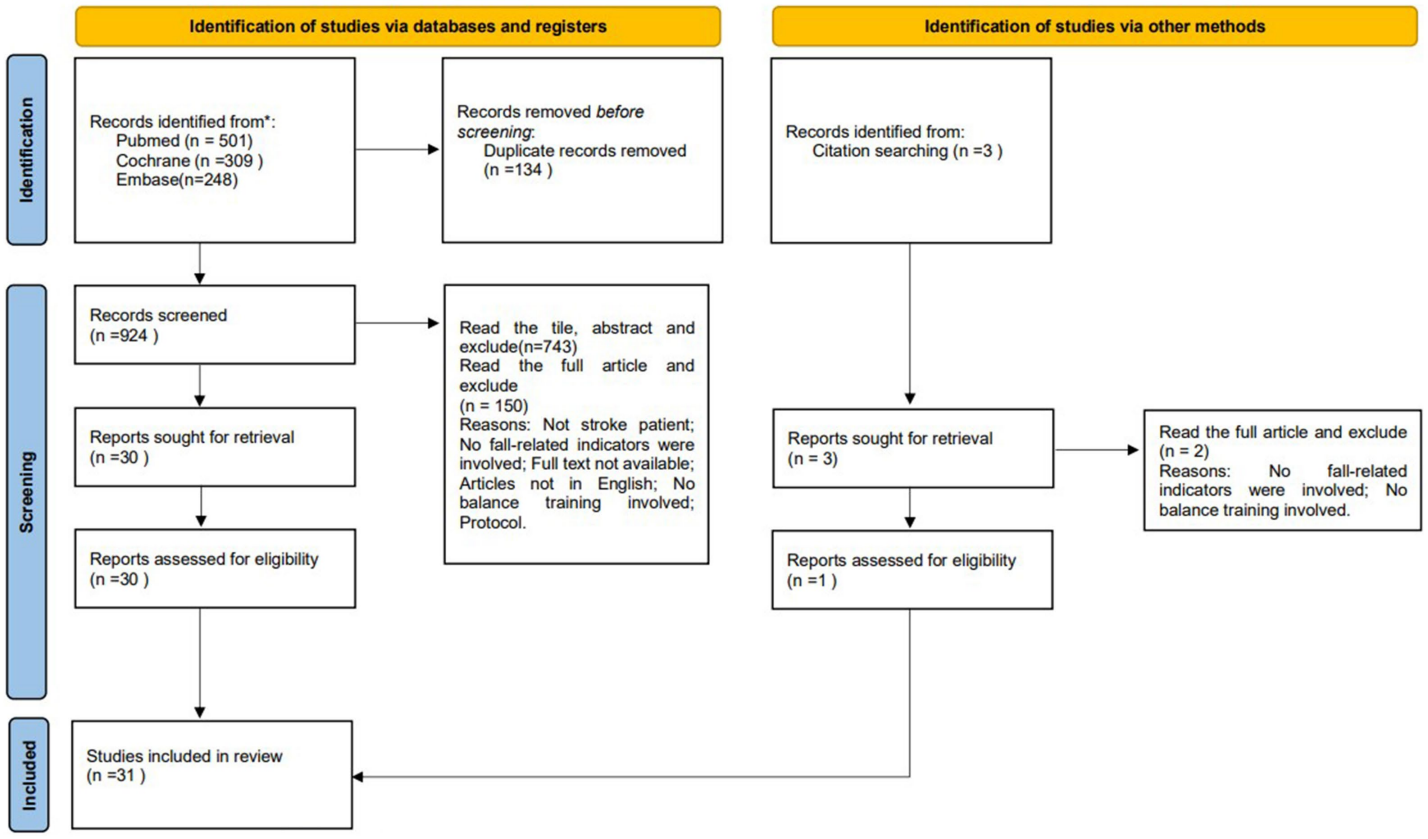
PRISMA 2020 flow diagram for new systematic reviews which included searches of databases, registers and other sources. *Consider, if feasible to do so, reporting the number of records identified from each database or register searched (rather than the total number across all databases/registers). **If automation tools were used, indicate how many records were excluded by a human and how many were excluded by automation tools. From: Page MJ, McKenzie JE, Bossuyt PM, Boutron I, Mulrow CD, et al. The PRISMA 2020 statement: an updated guideline for reporting systematic reviews. BMJ 2021; 372:n71. doi: 10.1136/bmj.n71. For more information, visit: http://www.prisma-statement.org/.

Thousand fifty-eight articles (PUBMED 501, Cochrane 309, and EMBASE 248) were identified. After excluding duplicate articles, 924 records were screened, 181 articles were selected for full-text screening, and 30 articles were finally selected. Manual retrieval of 1 paper, a total of 31 articles were selected. The flow chart in Figure 1 shows the article selection procedure according to the PRISMA guidelines (14).

The papers were published between 2012 and 2023, of which 24 were published in the past 5 years (2018–2023). And the sample ranged from 8 to 100 adult patients with post-stroke symptoms. Twenty-six of the included studies were randomized controlled trials, other study designs were not randomized or controlled, Table 1 summarized the characteristics of the included studies.

**Table 1:** The characteristics of the included studies.

Author (Year)	Study Design	Country/Region	Stroke			Gender (men/women)	Age (EG/CG)	Sample (EG/CG)	Intervention			Outcomes		Follow-up
			Side	Type	Duration				EG	CG	Time	Primary	Secondary	
Junata (2021)	RCT	Hong Kong	L/R (15/15)	First unilateral Ischemic/hemorrhagic	>1 year	24/6	≥50 (60.6 ± 5.5/60.1 ± 5.8)	30 (16/14)	Rapid movement training (RMT) The Kinect-based rapid movement training platform system	Conventional balance Training (CBT)		BBS*** TUG	FMA** ABC BI	
Kannan (2020)	Before and after	United States	L/R (6/5)	Chronicity Cortical Ischemic/hemorrhagic (4/7)	>6 months (9.63 ± 6.63)	6/5	60.63 ± 4.24	11	Exercise-based conventional training (EBCT)		6 weeks	Reactive balance control Volitional balance control* MVL△ Fall↓	BBS** TUG** FSST*** 6MWT** LST** CST***	
Okonkwo (2018)	Quasi-experimental	Multi-center		First Sub-acute ischemic	3–6 months	45/55	30–65 (53.94 ± 9.316/49.30 ± 12.214)	100 (50/50)	Task-specific balance training (TSBT) Cognitive impaired group (CIG)	Task-specific balance training (TSBT) Non-cognitive impaired group (NCIG)	12 months	BBS***		4 months*** 8 months*** 12 months***
Liu (2018)	RCT	Hong kong	L/R	Ischemic/hemorrhagic	1–6 years	57/32	55–85 (60.47 ± 5.61/60.46 ± 5.91)	89 (45/44)	Cognitive behavior therapy (CBT) + Task-oriented balance training (TOBT)	General health education (GHE) + TOBT		ABC**	SAFE*** BBS** LADL***	
Zhao (2022)	RCT	China	L/R	One-side hemiparesis Ischemic/hemorrhagic	6 months	30/10	18–75 (60.4 ± 12.32/54.45 ± 13.94)	40 (20/20)	Gaze stabilization exercises (GSEs) + physical therapy	physical therapy	4 weeks	BBS	TUGT △Gait characteristics swing phase (SW)* absolute symmetry index (ASI)* △Plantar pressure*	
Correia (2021)	RCT	Portugal	L/R	Ischemic/Hemorrhagic	3–15 months	46/22	> 60 73(60–87)/73 (61–87)	68 (33/35)	oculomotor and gaze stability exercises+ usual rehabilitation program;	usual rehabilitation program	3 weeks	Fall rates (11.4%/0)	BBS*** TUG***	
Kim (2022)	RCT	Korea	L/R	chronic stroke	>6 months	19/11	62.53 ± 10.54/60.67 ± 10.04	30 (15/15)	traditional stroke rehabilitation program and trunk stabilization exercises using laser pointer visual feedback	traditional stroke rehabilitation program and trunk stabilization without visual feedback	6 weeks	BBS*** △static plantar pressure*** △dynamic plantar pressure*** 10 MWT*** K-FES***		
Liao (2018)	RCT	TAIWAN	L/R	Ischemic/Hemorrhagic	>6 months	38/18	59.08 ± 9.16	56 (19/18/19)	BT: balance training (the weight shift training using the Biodex Balance System, visual biofeedback balance training) LW: lateral wedge (5° lateral wedge insole placed in the shoe)	Usual rehabilitation program	6 weeks	△balance CAT***	TUG*	10-week* 18-week*
Yu (2020)	RCT	CHINA	L/R	hemorrhage or infarction	≥3 months	41/30	30–75 (63.03 ± 8.92/ 58.69 ± 9.72)	71 (35/36)	(BWS-TC) Body weight support (BWS) treadmill training - Tai Chi (TC) footwork training and conventional rehabilitation therapies	conventional rehabilitation therapies	12 Weeks	△LOS test*	△Gait Analysis* FMA BBS	
Hwang (2019)	A mixed-method design	Korea	L/R	hemiplegic	>1 year	4/6	40–80	10	Tai Chi based stroke rehabilitation program		12-month	SAR test* BBS** TUG ADL		
Huang (2019)	RCT	CHINA	L/R	Ischemic/Hemorrhagic	11.36(4.91)/10.50(4.24) months	22/6	30–75 (62.21 ± 9.74/ 59.93 ± 9.96)	28 (14/14)	The BWS-TC footwork training rehabilitation program	conventional rehabilitation programs	12 Weeks	△LOS*	m-CTSIB* FRI↓ FMA↑	
Schmid (2012)	RCT	American	unknown	chronic stroke	>6 months	38/9	≥18 63.1 ± 8.8	47 (10/37)	group yoga yoga-plus (group yoga plus at-home yoga/relaxation audio recording)	Wait-List usual care	8 weeks	mRS BBS ABC FOF		
Komiya (2021)	RCT	Japan	unknown	ischemic or hemorrhagic	≥12 months	15/7	75.0 ± 11.5	30 (15/15)	Exercise by Real-Time Postural Feedback System; using the stabilometer with in-built disturbance generation	stand on the polyurethane mat; used a polyurethane mat	six weeks	TUG**	△SPPB m-GES* FES	10 weeks↑
Chun (2016)	RCT	Korea	L/R	infarction, hemorrhage	≥6 months	18/10	56.21 ± 9.3/53.93 ± 9.21	28 (14/14)	The Spine Balance 3D system	Biodex Balance System	7 weeks	BBS*** 10mWT*** TUG*** FRT*** KFES-I** △LOS		
Brunelli (2020)	RCT	Italy	L/R	ischemic or hemorrhagic	within 4 weeks	13/11	58.1 (20.4) /59.7 (14.2)	24 (12/12)	Computerized Balance Training (CBT) + conventional physiotherapy biodex balance system	conventional physiotherapy	4 Weeks	BBS***	TBS* 2MWT** BI CTSIB*	
Hong (2020)	RCT	South Korea	L/R	unkonwn	≥6 months 19 ± 8.38/15.33 ± 7.47	10/7	56.63 ± 8.78/66.22 ± 11.55	17 (8/9)	the cognitive task group (CBT)	the general task group (GBT)		TUG* BBS Gait		
Saleh (2019)	RCT	Egypt	L/R	Infarction/ Hemorrhagic	6 months- 1 year	24/26	45–55 (49.53 ± 1.8/ 50 ± 1.96)	50 (25/25)	dual task training in water	dual task training on land	6 Weeks	△OASI* △APSI* △MLSI**	△WS** △Step length* △Time of support***	
Ku (2020)	RCT	Taiwan	L/R	ischemic/hemorrhagic/mixed	≥6 months	14/6	20–80 55 (7.3)/52.5 (6.3)	20 (10/10)	Ai Chi water-based exercise	conventional water-based exercise	6 Weeks	LOS*	BBS* FMA*	
Furnari (2014)	RCT	Italy	L/R	infarction	6.3 ± 1.4 months	20/20	70 ± 6	40 (20/20)	Hydrokinesy therapy	conventional physical therapy	8 weeks	TT***	BI***	
Temperoni (2020)	RCT	Italy	L/R	unilateral hemiplegia chronic	>6 months	21/12	52.44 ± 10.51/52.01 ± 17.1	28 (15/13)	water-based sequential preparatory approach (SPA) balance training	conventional aquatic therapy	4 Weeks	BBS*	MBI TBG	4 weeks
Aslam (2021)	RCT	PARKSTAN	L/R	Infarction/ Hemorrhagic	unknown	18/12	50–60	30 (15/15)	Exer-gaming group (EGG)	traditional training (TBT)	6 weeks	BBS*** TUG***		
Kannan (2019)	RCT	USA	L/R	Infarction/ Hemorrhagic	>6 months	13/11	57.5 ± 8.04/ 61 ± 4.6	24 (12/12)	Wii-fit games in conjunction with cognitive training	traditional training, standing and walking	6 Weeks	△LOS*	BBS*** TUG* 6MWT*** ABC	
Golla (2018)	RCT	German	L/R	Unknown	18.6 (3.8) weeks	7/4	≥60 74.0 (8.1)	11 (5/6)	Home-based balance training using Wii Fit	traditional training	12 Weeks	BBS △Dynamic Gait Index ABC TUG		6 weeks↑ 12 weeks↑
Subramaniam (2014)	Before and after	America	L/R	ischemic/hemorrhagic	>6 months	4/4	28–65	8	Wii Fit+ cognitive training		5 days	△LOS*	BBS*** TUG***	
Cho (2012)	RCT	Korea	L/R	Infarction/ Hemorrhagic	>6 months	14/8	65.26(8.35) 63.13(6.87)	22 (11/11)	virtual reality balance training by using the balance board game system (Wii Fit)	traditional training	6 weeks	△Postural sway velocity	BBS** TUG***	
Hung (2016)	RCT	TaiWan	L/R	Infarction/ Hemorrhagic	≥6 months	16/7	>18 52.75/55.20	23 (12/11)	Tetrax biofeedback video games	traditional training	6 weeks	TUG*** FR**		
Inoue (2022)	RCT	Japan	L/R	Infarction/ Hemorrhagic early subacute phase	1 week to 3 months	25/12	40–80 61.6 (10.1)/ 63.1 (10.1)/ 69.7 (8.7)	57 (18/19/20)	BEAR group:robotic balance training and conventional inpatient rehabilitation IBT group: robotic balance training and conventional inpatient rehabilitation (BEAR group)	conventional inpatient rehabilitation-only (CR group)	2 weeks	Mini-BESTest*	TUG* FES	2 weeks*
Schinkel (2019)	RCT	Canada	L/R	unknown	>6-months	12/4	66.1 (8.3)/ 60.3 (8.9)	8 (4/4)	perturbation-based balance training (PBT)	traditional balance training (TBT)	6 weeks	△reactive stepping characteristics*		6 months↑*
Handelzalts (2019)	RCT	Israeli	L/R	first unilateral ischemic/hemorrhagic	43.7 (19.7)/ 40.3 (18.1) days	24/8	60.4 (10.1)/ 62.5 (8.4)	32 (16/16)	Perturbation-based balance training (PBBT)	weight shifting and gait training (WS&GT)	2.5 weeks	△Multiple-Step Threshold	BBS 10MWT 6MWT ABC	5 weeks
Mansfield (2017)	prospective cohort study	Canada	L/R/both	Unknown sub-acute	53.6 (21.0)/ 53.4 (19.2) days	46/16	60.1 (15.3)/ 58.8 (9.6)	62 (31/31)	Perturbation-based balance training (PBT)	historical control group (HIS)	3 weeks	FRI↓	BBS*** TUG*	
ChoH (2020)	RCT	Korea	L/R	Infarction/ intracerebral hemorrhage	>6 30.33(7.69)/26.13(6.58) months	20/10	55.67(7.62)/ 59.07(5.62)	30 (15/15)	audiovisual action observation (AAO) training	visual action observation training (VAO)	8 weeks	△Balance index*	FRI*	

*p < 0.05, **p < 0.01, ***p < 0.001. ↑, Rise; ↓, Decline. Experimental group/control group (EG/CG). Functional outcomes: BBS, Berg Balance Scale; TUG/TUGT, Timed-Up-and-Go Test; FMA, Fugl-Meyer Assessment; ABC, Activities Balance Confidence Scale; BI, Barthel Index for Activities of Daily Living; FSST, Four Step Square Test; 6MWT, Six-Minute Walk Test; LST, Lateral Step Test; CST, 30 s Chair Stand Test; 10MWT: 10-m walk test; K-FES, Korean version of the Fall Efficacy Scale scores; SAR, sit-and-reach test; m-CTSIB, Modified Clinical Test of Sensory Integration of Balance; FRI, Fall Risk Index assessment; FR, Forward Reach; TBS, Tinetti Balance Scale; SAFE, Survey of Activities and Fear of Falling in the Elderly; IADL, Lawton instrumental activities of daily living scale; m-GES, the modified Gait Efficacy Scale; FES, Fall Efficacy Scale; FRT, Functional Reach Tests; KFES-I, The Korean version of the Fall Efficacy Scale-International; 2MTW, 2-Minute Walk Test; TT, Tinetti Test. Biomechanical outcomes △: LOS, Limits of Stability (LOS) test; SPT, Stance Perturbation Test; MVL, Movement Velocity; COP, center of pressure; CTSIB, Clinical Test of Sensory Integration and Balance; Balance CAT, computerized adaptive test; m-BEST Mini-Balance Evaluation Systems Test; SPPB, Short Physical Performance Battery; OASI, Overall Stability Index; APSI, Anteroposterior Stability Index; MLSI, Mediolateral Stability Index; WS, Walking speed.

**Figure 2:**
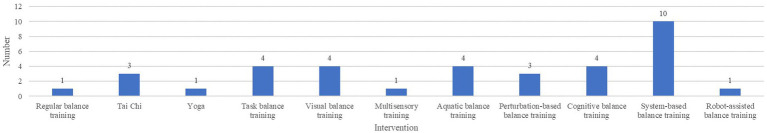
Number of articles on each type of intervention.

Among the interventions in the 31 studies, one study selected regular balance training, three studies selected Tai chi, one study selected yoga, four studies selected task-balance training, four studies selected visual balance training, one study selected multisensory training, four studies selected aquatic balance training, three studies selected perturbation-based training, four studies selected cognitive balance training, 10 studies selected system-based balance training, one study selected robot-assisted balance training. Interventions of the articles are shown in Table 2 and Figure 2.

**Table 2:** Interventions of the articles.

**Intervention**			**Article**	**Number**	**Sample**
Balance training only	Regular balance training	Exercise-based conventional training (EBCT)	Kannan (2020)	1	11
	Tai Chi	Body weight support- Tai Chi (BWS-TC)	Yu (2020)^(1)^	3	35
			Huang (2019)^(1)^		14
		Tai Chi based stroke rehabilitation program	Hwang (2019)		10
	Yoga	yoga-plus (group yoga plus at-home yoga/relaxation audio recording)	Schmid (2012)	1	10
Combined balance training and other interventions	Task balance training	Task-specific balance training (TSBT)	Okonkwo (2018)	4	100
		Task-oriented balance training (TOBT)^③^	Liu (2019)		89
		Task of moving the lower extremity of the affected side^④^	Hong (2020)		9
		The motor dual task training^⑤^	Saleh (2019)		25
	Visual balance training	Gaze stabilization exercises (GSEs)	Zhao (2022)	4	20
		Oculomotor and gaze stability exercises	Correia (2021)		33
		Trunk stabilization exercises using laser pointer visual feedback	Kim (2022)		15
		Visual biofeedback balance training	Liao (2018)		19
	Multisensory training	Audiovisual action observation (AAO) training	ChoH (2020)	1	15
	Aquatic balance training	Dual task training in water^⑤^	Saleh (2019)	4	25
		Ai Chi water-based exercise	Ku (2020)		10
		Hydrokinesy therapy	Furnari (2014)		20
		water-based sequential preparatory approach (SPA) balance training	Temperoni (2020)		15
	Perturbation-based balance training (PBT/PBBT)		Schinkel (2019)^(2)^	3	8
			Mansfield (2017)^(2)^		31
			Handelzalts (2019)^(2)^		16
	Cognitive balance training	Balance and cognition training using traffic signals^④^	Hong (2020)	4	8
		Cognitive-motor interference^①^	Subramaniam (2014)		8
		Cognitive behavior therapy (CBT)^③^	Liu (2019)		45
		Cognitive-Balance Control Training^⑥^	Kannan (2019)		12
	System-based balance training	The Kinect-based rapid movement training (RMT) platform system	Junata (2021)	10	16
		Real-Time Postural Feedback System	Komiya (2021)		11
		The Spine Balance 3D system^②^	Chun (2016)		14
		Biodex Balance System^②^			14
		Computerized Balance Training (CBT), Biodex Balance System	Brunelli (2020)		12
		Kinect-based exer-gaming training	Aslam (2021)		15
		Wii-fit games in conjunction with cognitive training^⑥^	Kannan (2019)		12
		Home-based balance training using Wii Fit	Golla (2018)		5
		Wii Fit^①^	Subramaniam (2014)		8
		Virtual reality balance training by Wii Fit	Cho (2012)		11
		Tetrax biofeedback video games	Hung (2016)		12
	Robot-assisted balance training	Balance Exercise Assist Robot (BEAR) training	Inoue (2022)	1	18

The same serial numbers ①-⑥ indicate in the same article. The same serial numbers (1)–(2) indicate the same type of intervention.

The results of 31 studies showed that the balance ability of stroke patients was improved, and the incidence of falls was reduced after balance training. Most of the outcome measures were the incidence of falls and indicators closely related to falls such as Berg balance scale (BBS), Timed Up-and-Go Test (TUG), Fall Risk Index (FRI), Fall Efficacy Scale score (FES), etc. In addition, some studies also use the instrumented balance tests, such as Biodex balance system, platform and other instruments to measure.

Most of the studies focused on patients after 6–12 months from the onset of stroke, requiring patients to be able to walk more than 10 meters independently or stand for more than 5 min. And there is a lack of researches for such stroke patients with early balance training, which may have better recovery effects (19). However, considering the safety of patients and potential medical disputes, it is often difficult to achieve, so robot-assisted balance training has unique advantages for early intervention of such patients (20).

The sample sizes of the interventions were generally small, with 23 studies having sample sizes of less than 50 and 8 studies having sample sizes of 50–100, suggesting that studies with larger sample sizes should be conducted. There was also a lack of multi-center studies, and only one was a multi-center study (21).

Six studies used integrated balanced-training approaches, two were cognitive and task balance training (22, 23), two were cognitive and Wii Fit balance training (24, 25), one was task and aquatic balance training (26), one was Audiovisual action observation (AAO) training (27). The results showed that a combination of multiple interventions improved the balance ability of stroke patients and reduced the incidence of falls compared to a single intervention measure. At present, there are few studies used multiple balance training. It is suggested that cognitive balance training, multi-sensory training, action observation training, exercise training, and other methods can be combined to achieve better results.

The mean age of the samples was between 50 and 75 years old. This shows that the participants were generally middle-aged and elderly. The majority of participants were male, accounting for about 63.5% (male 704/female 405).

Two of the articles used before and after design, one article used quasi-experimental study, one article used a prospective cohort study, one article used a mixed-method design (including qualitative analysis), and the others were randomized controlled trials. Two articles covered acute stroke (<1 month), 11 articles covered early stroke (1–3 months) and sub-acute stroke (3–6 months), and 21(21/31, 67.7%) articles were chronic stroke (>6 months). A total of 8 articles carried out subsequent follow-up, accounting for about 25.81% (8/31), and the follow-up duration ranged from 2 weeks to 12 months. Only one study was home-based and conducted unsupervised training after 6 weeks of training independently at home (28). Fear of falling is a major psychological disorder in stroke patients that may limit patients’ participation in daily activities and functional training, four studies (11, 23, 29, 30) showed that through balance training can enhance the balance confidence of stroke patients.

## 4 Discussion

This scoping review summarized all the available primary evidence for preventing falls after stroke based on balance training, including single and integrated balance training. It was found that balance training can improve the balance function and confidence of stroke patients, reduce the fear of falling and incidence of falls. In general, most of the studies focused on middle-aged and older people, and there was a lack of studies on young people, so studies in the future can compare the effects of balance training in different age groups. The research subjects of the study were mainly chronic stroke, lack of researches involved early and sub-acute stroke. It is suggested that more research should focus on the early rehabilitation stage of stroke in the future.

Only 25.81% of the studies involved follow-up, multi-stage evaluation through follow-up is more objective and instructive. Most studies do not have follow-up or the follow-up time is particularly short. It is recommended to increase and conduct long-term follow-up through big data platforms after intervention to observe the long-term occurrence of falls in patients. In addition, the outcomes before, during, and after the intervention can be evaluated and compared to analyze the effect of the intervention, and the model can also be constructed to predict the occurrence of falls by collecting these data.

Four studies showed through balance training, improved the willingness of patients to walk, and help patients to training, but it may also lead to overconfidence of patients, bringing some potential risks. Therefore, they should be monitored by professionals training process, equipped with emergency, in case of falling and other situations that patients can be rescued in time. A study showed that patients had discomfort such as pain in the early stage of training (31). Therefore, it is recommended to carry out preparatory activities and stretching exercises before exercise training, and the intensity of training should be increased slowly and gradually to avoid pain and other discomfort symptoms.

Among these 31 articles, the most published intervention was System-based balance training, especially Wii Fit, which became a hot research topic and efficiently helped patients complete balance training through games. The second is task, Visual, cognitive and aquatic balance training, all of them were 4. The balance training combining task and cognitive is a research hotspot; aquatic balance training limits its development due to application scenario limitations. The number of papers in Balance training only was small, ranging from 1 to 3, it may be that the effect of a single training method was not as good as that of comprehensive training. There was only one article on Multisensory training, which requires more research in the future. There was only one article on Robot-assisted balance training, however, balance training is a long-term process, robot-assisted balance training makes patients’ home training more secure, and it is suggested that more applications should be applied in future research.

### 4.1 Balance training only

#### 4.1.1 Regular balance training

There was only one article about regular balance training, and the study showed that, exercise-based conventional training (EBCT) can significantly increase the limits of Stability (LOS) and reduce the fall incidence. In fact, regular balance training only needs some simple tools to complete, such as stools, dumbbell, training ball, etc. Training is less costly and simple, but needs to be tailored to provide a comparable level of challenge for everyone (32).

#### 4.1.2 Tai Chi

Three studies have shown that Taijiquan can improve the balance function of stroke patients. The training is generally 12 weeks, and can be as long as 12 months, training 2–5 times per week, each time is 40–60 min (31, 33, 34). In Yu’s study (33), the intervention in the experimental group included body weight support (BWS) treadmill training - Tai Chi (TC) footwork training, and conventional rehabilitation therapies; the control group only conventional rehabilitation therapies, for the combination of a variety interventions, it is still necessary to explore the effect of single intervention by increasing groups (three or four groups). Two studies combined weight support with tai chi. BWS gait training focused on improving sagittal function, while TC required subjects to perform symmetric and diagonal movements. Moreover, BWS could help stroke patients recover as early as possible without worrying about falls (33, 34). By used quantitative analysis (31), The results of a small 12-month qualitative study showed that a modified Tai Chi program is safe and feasible, and it expected to improve functional and balance outcomes related to fall prevention in stroke survivors (31). It is suggested that more articles should use both qualitative and quantitative methods to evaluate the effect in the future.

#### 4.1.3 Yoga

A study showed (29) that 8 weeks of yoga, which included sitting, standing, and floor poses for relaxation and meditation, reduced patients’ fear of falling, improved balance ability and confidence. However, the sample size of the study was small.

### 4.2 Combined balance training and other interventions

#### 4.2.1 Task balance training

Four articles were task balance training, 3 of them were related to “cognitive.” Okonkwo’s study was treated using the task-specific activities parameters targeted at optimizing balance, the results showed improved balance control and more effective in cognitive impaired (CI) (21). However, more researches were needed to verify the effect of task balance training in CI patients. In Liu’s study, cognitive behavior therapy (CBT) + task-oriented balance (TOBT), which underwent 90-min interventions 2 days per week for 8 weeks, significantly reduced patients’ fear of falling and improvements in balance and independent daily living (23). Hong’s study showed that cognitive task (CBT) was more effective intervention compared with general task group (GBT) to improve balance and gait ability of stroke patients (22). A study used the motor dual task training to improve balance including walking, holding a ball, standing on a balance board (26). The training session lasted for 45 min including warm up exercise, main exercise, cool down exercise, the training mode of this design is worth learning.

#### 4.2.2 Visual balance training

Four papers used eye movements for balance training, including gaze stability training and visual feedback balance training. Two papers used gaze stability training by moving the eye, target, head, head and target horizontally and vertically, 5–7 days a week, twice a day for about 30 min each time (35, 36). Two papers using visual feedback balance training, participants were instructed to hit a target with the laser pointer and move to a specified location (8 directions included: Front, back, left, right, left oblique before, right oblique before, left oblique after and right oblique after) for 20–30 min, three times a week for 6 weeks (37, 38).

Two studies have shown (35, 36) that gaze stability training can improve the BBS score of patients and reduce the incidence of falls. BBS score is widely used to assess the risk of falls, and patients with a BBS score of 46 or lower have a higher probability of falls (39); 2 studies have shown (37, 38) that the use of visual feedback based balance training can improve the balance ability of chronic stroke patients and reduce the incidence of falls.

#### 4.2.3 Multisensory training

A study was multisensory training, Audio-visual Action Observation Training (AAO) (27) was a combination of rhythmic auditory stimulation, visual training, and action observation training. The intervention consisted of 3 min of action observation training and rhythmic auditory stimulation, 12 min of physical training, three times a week for 8 weeks. Compared with the group receiving action observation training only, AAO combined with vision and hearing can effectively improve the balance ability of patients and reduce the incidence of falls.

#### 4.2.4 Aquatic balance training

Four studies used aquatic balance training for fall prevention in stroke patients (26, 40–42). A study combined dual task training and aquatic and showed aquatic motor dual task training is more effective in improving balance and gait in chronic stroke patients than land (26). One study showed that Ai Chi was superior to traditional water-based exercise in improving balance function in stroke patients, it consists of 16 movements, including breathing, upper limb movements, lower limb movements, trunk control and coordination exercises, 60 min each time, 3 times a week for 6 weeks (40). One Study used intensive hydrokinesitherapy consisting of (warm-up exercises, the Halliwick method, Tai chi, etc.),1 h per session, 3 times a week for 8 weeks, patients who received hydrokinesitherapy showed more significant improvement than those who received conventional training (41). A study used a water-based sequential preparatory approach (SPA) compared with traditional aquatic therapy and showed SPA was more effective for balance rehabilitation (42).

#### 4.2.5 Perturbation-based balance training

Three studies used perturbation-based balance training. All studies showed that perturbation-based training enhanced balance function and reduced the incidence of falls in stroke patients, which supports using PBT in balance training programs poststroke (30, 43, 44). One study showed that PBT could enhance the balance confidence of patients (30).

#### 4.2.6 Cognitive balance training

Three studies used cognitive balance training. Hong’s study showed the effects of cognitive task training, performed 30 min a day, three times a week for 4 weeks, showed significant improvement in walking and balancing abilities after intervention (22). A study was cognitive-Balance Control Training, demonstrated good adherence and evidence reducing cognitive-motor interference and improving balance control in stroke survivors (25). Future studies could examine the effects and long-term changes of such a dual-task (DT) training paradigm applied to improve fall efficacy. In Liu’s study, cognitive behavior therapy augmented the beneficial effects of task-oriented balance training (TOBT) in reducing the fear of falling in chronic stroke survivors (23).

#### 4.2.7 System-based balance training

In recent years, new technology had emerged to compensate the shortcomings of traditional rehabilitation. System-based training, such as virtual reality, platform, and game, have been well applied in balance training for stroke patients. A total of 10 studies included system-based balance training.

Two study used the Kinect-based rapid movement training platform to prompt the rapid movement training (RMT)/exer-gaming training (EGT), showed it provided beneficial effects on balance function, improving dynamic balance and mobility (11, 45). In Komiya’s study (46), used 6-week balance exercise by Real-Time Postural Feedback System, confer a positive effect on the walking ability in patients with chronic stroke and increase their self-confidence in gait performance. A study (47) used the newly developed Spine Balance 3D system to compare with the well-known Biodex Balance system, Spine Balance 3D system can perform eight directions in 3D space (front, back, left, right and diagonal). The results showed the 3D spine balance system was more effective than the traditional 2D balance training system in gait and dynamic balance rehabilitation. In Brunelli’s study (48), used Computerized Balance Training (CBT) on Biodex Balance System, once a day, five times a week for 4 weeks, showed that early computerized balance training is an effective therapeutic tool to improve balance and gait endurance in patients with subacute stroke. Four studies used Wii Fit improved balance control in stroke survivors (24, 25, 28, 49), future studies should examine the dose–response effects and long-term changes of it applied to improve fall efficacy. A study used the Tetrax biofeedback system, showed it is a feasible adjunctive program (50).

#### 4.2.8 Robot-assisted balance training

Robot-assisted rehabilitation of stroke patients holds excellent promise. One study with BEAR (Robot for Balance Exercise Assisted), interventions for 2 weeks, assessment before and after the intervention, and at 2 weeks follow-up, improved balance in patients with subacute stroke (20). Robot-assisted training can improve the safety of home training for patients; in the future, it has great application potential in early/subacute stroke patients with hemiplegia.

### 4.3 Limitations

This study has the following limitations. First, this study was a scoping review and failed to evaluate the data systematically. Secondly, due to the heterogeneity of the evidence, data integration and Meta-analysis cannot be performed at present. Finally, only articles published in English were included.

### 4.4 Implications

The sample size of studies is generally small, and large-scale multi-center RCT experiments are recommended. Long-term follow-up is recommended to observe the effect of exercise training. It is suggested that remote balance training intervention should be carried out if conditions permit, so that more people can enjoy cheap and convenient medical services. Future studies should combine various intervention methods, because falls caused by balance dysfunction may result from a combination of factors. Therefore, comprehensive balance exercise training methods are recommended. In the process of balance training, how to ensure the safety of patients, including the protection of training equipment and the safety of training process, as well as how to rescue patients in the process of emergency is worthy of further consideration.

This review collected all the studies on preventing falls in stroke patients by balance training. Balance training can improve the balance ability of stroke patients and reduce the incidence of falls. This study provides a reference for balance training and fall prevention in stroke patients, and researchers can combine these balance training methods to maximize the advantages.

## Data availability statement

The original contributions presented in the study are included in the article/Supplementary material, further inquiries can be directed to the corresponding author.

## Author contributions

SZ and KC: conceptualization. SZ, KC, and ZL: data curation. SZ, KC, and ZL: formal analysis. KC: methodology and writing – original draft. SZ and ZL: supervision. SZ, KC, ZL, YT, and FL: writing – review and editing. All authors contributed to the article and approved the submitted version.
