# Supplementary material for: Advances in balance training to prevent falls in stroke patients: a scoping review
Source: Front Neurol. 2024 Feb 5;15:1167954. doi: 10.3389/fneur.2024.1167954 (PMC10875131; doi:10.3389/fneur.2024.1167954)
Supplement: Supplementary file 1 [file Data_Sheet_1.DOCX]

***Supplementary Material***

**Advances in balance training to prevent falls in stroke patients: a scoping review**

**Siyi Zhu, Kehan Chen, Zuoyan Liu*, Yidan Tang, Fuxia Lan**

***Correspondence: Zuoyan Liu,** **Zuo.yan.2008@163.com**

**Appendix 1: Detailed Electronic Search Strategy**

**Search methods**

**PubMed**

13/1/2023

501 Results

No limits applied

(((((((((((stroke[MeSH Terms])) OR ("cerebral stroke")) OR ("ischemic stroke")) OR ("cerebrovascular accident")) OR ("cerebral vascular event")) OR ("transient ischaemic attack")) OR ("post stroke")) OR ("after stroke")) AND ((((((((((((Postural Balance[MeSH Terms]) OR (Balance)) OR ("balance control")) OR ("center of pressure")) OR (" postural stability")) OR (" postural balance")) OR ("postural sway")) OR (stability)) OR ("static balance")) OR ("static stance")) OR ("dynamic balance")) OR (equilibrium))) AND ((((((Exercise[MeSH Terms]) OR (Train*)) OR (Physical)) OR (Activity)) OR ("Physical intervention")) OR ("Physical activity"))) AND ((((accidental falls[MeSH Terms]) OR (Fall*)) OR ("Fall prevention")) OR (slip))

**Embase**

13/1/2023

248 Results

No limits applied

#1 'cerebrovascular accident'/exp

#2 stroke:ti,ab,kw OR 'cerebral stroke':ti,ab,kw OR 'ischemic stroke':ti,ab,kw OR 'cerebrovascular accident':ti,ab,kw OR 'cerebral vascular event':ti,ab,kw OR 'transient ischaemic attack':ti,ab,kw OR 'post stroke':ti,ab,kw OR 'after stroke':ti,ab,kw

#3 #1 OR #2

#4 'body equilibrium'/exp

#5 balance:ti,ab,kw OR 'balance control':ti,ab,kw OR 'center of pressure':ti,ab,kw OR 'postural stability':ti,ab,kw OR 'postural balance':ti,ab,kw OR 'postural sway':ti,ab,kw OR stability:ti,ab,kw OR 'static balance':ti,ab,kw OR 'static stance':ti,ab,kw OR 'dynamic balance':ti,ab,kw OR equilibrium:ti,ab,kw

#6 #4 OR #5

#7 'exercise'/exp

#8 train*:ti,ab,kw OR physical:ti,ab,kw OR activity:ti,ab,kw OR 'physical intervention':ti,ab,kw OR 'physical activity':ti,ab,kw

#9 #7 OR #8

#10 'falling'/exp

#11 'accidental falls':ti,ab,kw OR 'fall prevention':ti,ab,kw OR slip:ti,ab,kw

#12 #10 OR #11

#13 #3 AND #6 AND #9 AND #12

**Cochrane**

13/1/2023

309 Results

No limits applied

#1 MeSH descriptor: [stroke] explode all trees

#2 ("cerebral stroke"):ti,ab,kw OR ("ischemic stroke"):ti,ab,kw OR ("cerebrovascular accident"):ti,ab,kw OR ("cerebral vascular event"):ti,ab,kw OR ("transient ischaemic attack"):ti,ab,kw (Word variations have been searched)

#3 ("post stroke"):ti,ab,kw OR ("after stroke"):ti,ab,kw

#4 #1 OR #2 OR #3

#5 MeSH descriptor: [Postural Balance] explode all trees

#6 (Balance):ti,ab,kw OR ("balance control"):ti,ab,kw OR ("center of pressure"):ti,ab,kw OR ("postural stability"):ti,ab,kw OR (" postural balance"):ti,ab,kw

#7 ("postural sway"):ti,ab,kw OR (stability):ti,ab,kw OR (" static balance"):ti,ab,kw OR ("static stance"):ti,ab,kw OR ("dynamic balance"):ti,ab,kw

#8 (equilibrium):ti,ab,kw

#9 #5 OR #6 OR #7 OR #8

#10 MeSH descriptor: [Postural Balance] explode all trees

#11 (Train*):ti,ab,kw OR (Physical):ti,ab,kw OR (Activity):ti,ab,kw OR ("Physical intervention"):ti,ab,kw OR ("Physical activity"):ti,ab,kw

#12 #10 OR #11

#13 MeSH descriptor: [Accidental Falls] explode all trees

#14 (fall*):ti,ab,kw OR ("Fall prevention"):ti,ab,kw OR (slip*):ti,ab,kw

#15 #13 OR #14

#16 #4 AND #9 AND #12 AND #15
